# Generation of protective immunity against *Orientia tsutsugamushi* infection by immunization with a zinc oxide nanoparticle combined with ScaA antigen

**DOI:** 10.1186/s12951-016-0229-2

**Published:** 2016-11-26

**Authors:** Na-Young Ha, Hyun Mu Shin, Prashant Sharma, Hyun Ah Cho, Chan-Ki Min, Hong-il Kim, Nguyen Thi Hai Yen, Jae-Seung Kang, Ik-Sang Kim, Myung-Sik Choi, Young Keun Kim, Nam-Hyuk Cho

**Affiliations:** 1 Department of Microbiology and Immunology, Seoul National University College of Medicine, 103 Daehak-ro, Jongno-gu, Seoul, 03080 Republic of Korea; 2 Department of Biomedical Sciences, Seoul National University College of Medicine, Seoul, Republic of Korea; 3 Institute of Endemic Disease, Seoul National University Medical Research Center and Bundang Hospital, Seoul, Republic of Korea; 4 Department of Materials Science and Engineering, Korea University, 145 Anam-ro, Seongbuk-gu, Seoul, 02841 Republic of Korea; 5 Department of Microbiology, Inha University School of Medicine, Incheon, Republic of Korea

**Keywords:** Zinc oxide nanoparticle, ZnO binding peptide, Scrub typhus, Vaccine

## Abstract

**Background:**

Zinc oxide nanoparticle (ZNP) has been applied in various biomedical fields. Here, we investigated the usage of ZNP as an antigen carrier for vaccine development by combining a high affinity peptide to ZNP.

**Results:**

A novel zinc oxide-binding peptide (ZBP), FPYPGGDA, with high affinity to ZNP (*K*_*a*_ = 2.26 × 10^6^ M^−1^) was isolated from a random peptide library and fused with a bacterial antigen, ScaA of *Orientia tsutsugamushi,* the causative agent of scrub typhus. The ZNP/ZBP-ScaA complex was efficiently phagocytosed by a dendritic cell line, DC2.4, in vitro and significantly enhanced anti-ScaA antibody responses in vivo compared to control groups. In addition, immunization with the ZNP/ZBP-ScaA complex promoted the generation of IFN-γ-secreting T cells in an antigen-dependent manner. Finally, we observed that ZNP/ZBP-ScaA immunization provided protective immunity against lethal challenge of *O. tsutsugamushi*, indicating that ZNP can be used as a potent adjuvant when complexed with ZBP-conjugated antigen.

**Conclusions:**

ZNPs possess good adjuvant potential as a vaccine carrier when combined with an antigen having a high affinity to ZNP. When complexed with ZBP-ScaA antigen, ZNPs could induce strong antibody responses as well as protective immunity against lethal challenges of *O. tsutsugamushi*. Therefore, application of ZNPs combined with a specific soluble antigen could be a promising strategy as a novel vaccine carrier system.

## Background

Biocompatible-nanomaterials exert an immunomodulatory effect on the immune system and engineered nanoparticles have been considered as promising adjuvants and/or carrier systems for vaccine development against infections and cancers [1, 2]. Zinc oxide (ZnO) nanoparticles (ZNPs), due to their good biocompatibility and low cost, have been widely applied as food ingredients, UV-blocking agents, and anti-microbial materials [3–5]. In addition, ZNPs possess promising potential for biomedical applications, such as bio-imaging and drug delivery [6]. In order to expand the applicability of ZNPs, diverse approaches have been used to explore the properties of peptides that enable binding to the surface of inorganic materials [7–9] and several types of peptides with high affinity to ZNPs have been identified [10–14]. Unlike covalently bound linker surface modifications, peptides bound to nanomaterials utilize high affinity non-covalent bonds, which simplify the process for functionalization of ZNPs.

Recent studies have reported that immune cells and organs are the primary sites for the deposition of inorganic NPs after systemic exposure, and NPs mediate inflammatory or immunomodulatory effects on innate and adaptive immune cells [15]. Our recent study in which mice were subcutaneously injected with iron oxide (Fe_3_O_4_)–zinc oxide (ZnO) core–shell nanoparticles also resulted in foreign body responses in the form of macrophage infiltration, but otherwise did not show any systemic distribution or toxicity at up to 200 mg kg^−1^ [16]. Nevertheless, ZNPs exposure might induce strong local inflammation at the injection site [17] and this can be linked to the generation of antigen-specific adaptive immune responses, including antibodies as well as T cell responses, when combined with a specific protein antigen [18]. Even though the detailed immunological mechanisms of how ZNPs stimulate the immune system and contribute to the generation of specific immunity against co-injected antigen need to be investigated [19], it is intriguing to observe that inflammatory responses induced by injection of ZNPs are linked to augmentation of antigen-specific adaptive immunity.

In order to investigate the potential applicability of ZNPs as a vaccine adjuvant and carrier system for infectious diseases, we selected scrub typhus, caused by *Orientia tsutsugamushi* infection, as a model disease. Scrub typhus is one of the main causes of acute febrile illness in the Asian-Pacific region [20, 21] and the rate of incidence has been estimated to be one million cases annually [22]. During the last decade, the incidence of scrub typhus has also rapidly increased in South Korea [23] and China [24]. In addition, sporadic outbreaks of scrub typhus in several countries in the endemic region make it a serious public health issue [25, 26]. Clinical symptoms of the mite-borne disease include eschar at the site of mite biting, lymphadenopathy, fever, headache, myalgia, and rash. Due to the lack of specificity of its early clinical presentation, delayed treatment with proper antibiotics, such as doxycycline or chloramphenicol, often leads to more severe organ failures, including acute respiratory distress, meningoencephalitis, gastrointestinal bleeding, acute renal failure, hypotensive shock, and coagulopathy [22]. However, an effective vaccine has not yet been developed despite continuous efforts in the last several decades [22]. While a major outer membrane protein, TSA56, has been studied as a conventional target for scrub typhus vaccine since it is an immunodominant antigen, many issues remain that need to be resolved for the development of an effective vaccine, especially for cross-protective immunity against diverse genotypes [22, 27]. Previously, our group reported the potential role of the ScaA protein, an autotransporter protein of *O. tsutsugamushi*, in bacterial pathogenesis and evaluated the immunogenicity of ScaA for protective immunity against lethal *O. tsutsugamushi* infection in mice, suggesting that ScaA should be considered as a novel target for scrub typhus vaccine [28, 29]. ScaA functions as a bacterial adhesion factor, and anti-ScaA antibody significantly neutralizes bacterial infection of host cells. In addition, immunization with ScaA not only provides protective immunity against lethal challenges with the homologous strain, but also confers significant protection against heterologous strains when combined with TSA56 [28].

In the present study, we screened and selected a high affinity ZBP and investigated whether ZBP conjugation with the bacterial antigen, ScaA, could further enhance the generation of adaptive immunity when complexed with ZNPs, by measuring antigen-specific humoral immunity as well as T cell responses. In addition, we also tested if ZNP/ZBP-ScaA complexes can provide protective immunity against lethal infections in vivo. Our results showed that immunization with ZNP/ZBP-ScaA complexes induced proper adaptive immune responses and could provide comparable protection against lethal challenges of *O. tsutsugamushi* as a conventional vaccine adjuvant, alum hydroxide, suggesting that ZNPs may potentially be used as an antigen carrier and adjuvant system when combined with ZBP-conjugated antigens.

## Results

### Preparation of ZnO nanoparticles

**Fig. 1:**
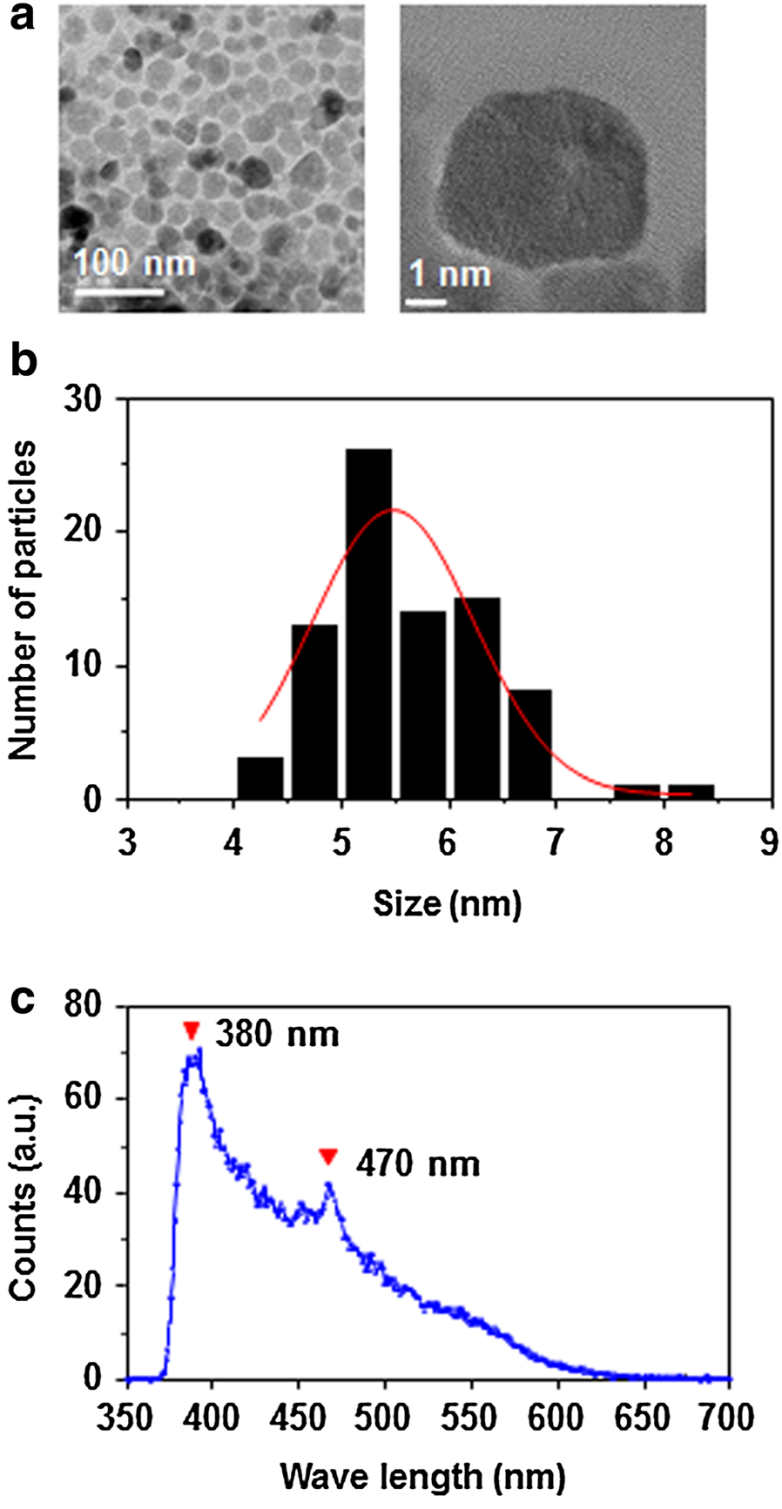
Characterization of ZnO nanoparticle (ZNP). **a** TEM images of the monodispered spherical ZNPs. **b** Gaussian size distribution of ZNPs. **c** Photoluminescence spectrum of ZNPs showing UV and visible emissions

The morphologies and particle sizes of the prepared ZNPs were observed by transmission electron microscopy (TEM) (Fig. 1a). ZNPs are almost spherically shaped. The size of ZNPs shows a Gaussian distribution and the nanoparticles have an average diameter and standard deviation of 5.48 ± 0.75 nm (Fig. 1b). The photoluminescence spectra of ZNPs under the excitation wavelength of 330 nm showed a major peak at ~380 nm, the expected emission of the ZnO bandgap (3.3 eV), as well as additional broad visible emissions with a peak at 470 nm (Fig. 1c), which were related to surface and defect emissions [30].

### Selection of novel ZnO-binding peptides

**Fig. 2:**
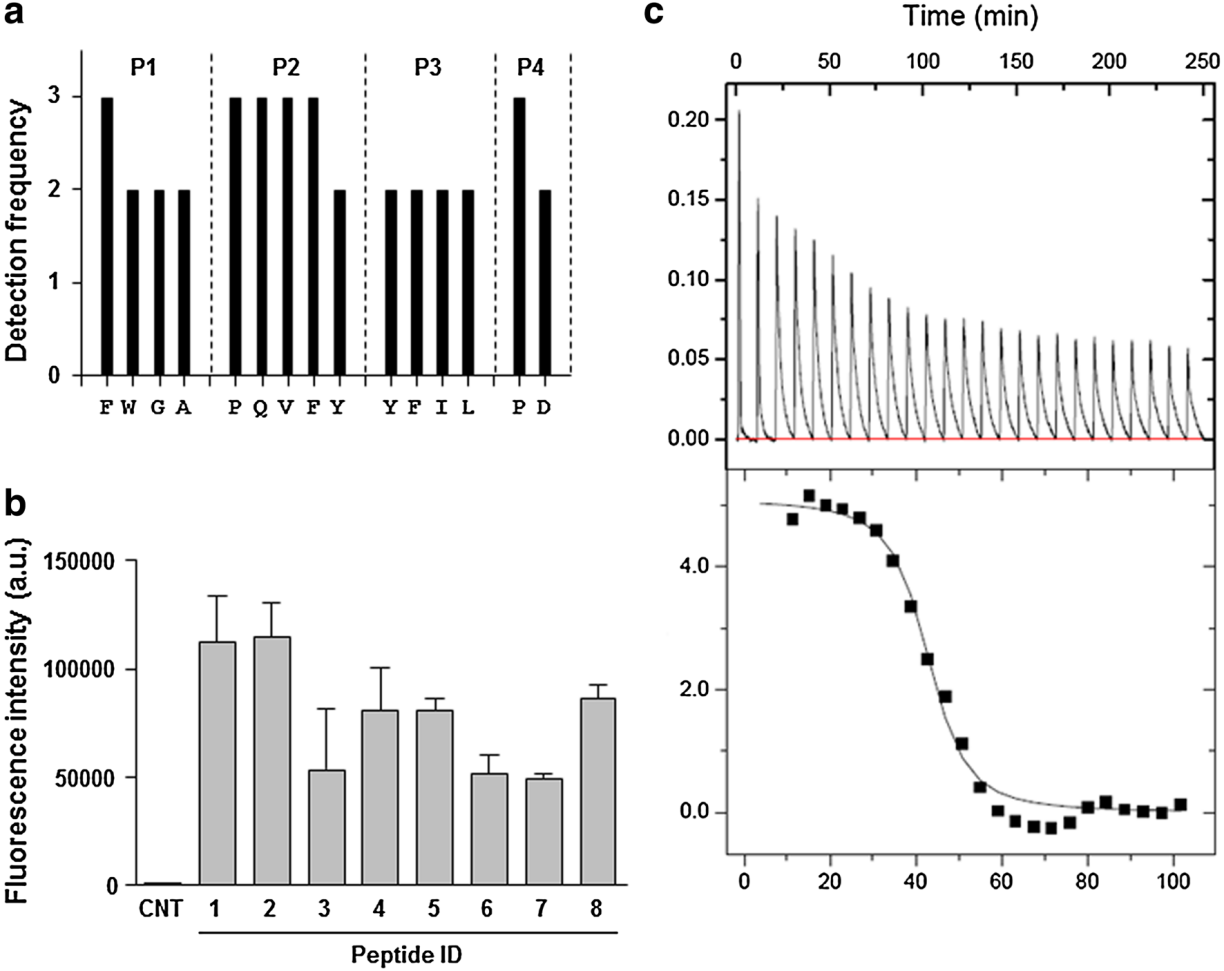
Screening and characterization of high affinity ZNP-binding peptide. **a** Amino acid sequences at the indicated positions (*P1*–*P4*) of peptides bound to ZNP were determined by mass spectrometry. **b** Relative affinities of selected peptides labeled with FITC were assessed by measuring fluorescent intensity of ZNP-peptide complexes. *Error bars* mean ± SD. **c** Detection of the interaction of a ZBP, FPYDGGDA, with ZNP by isothermal titration calorimetry (ITC)

**Fig. 3:**
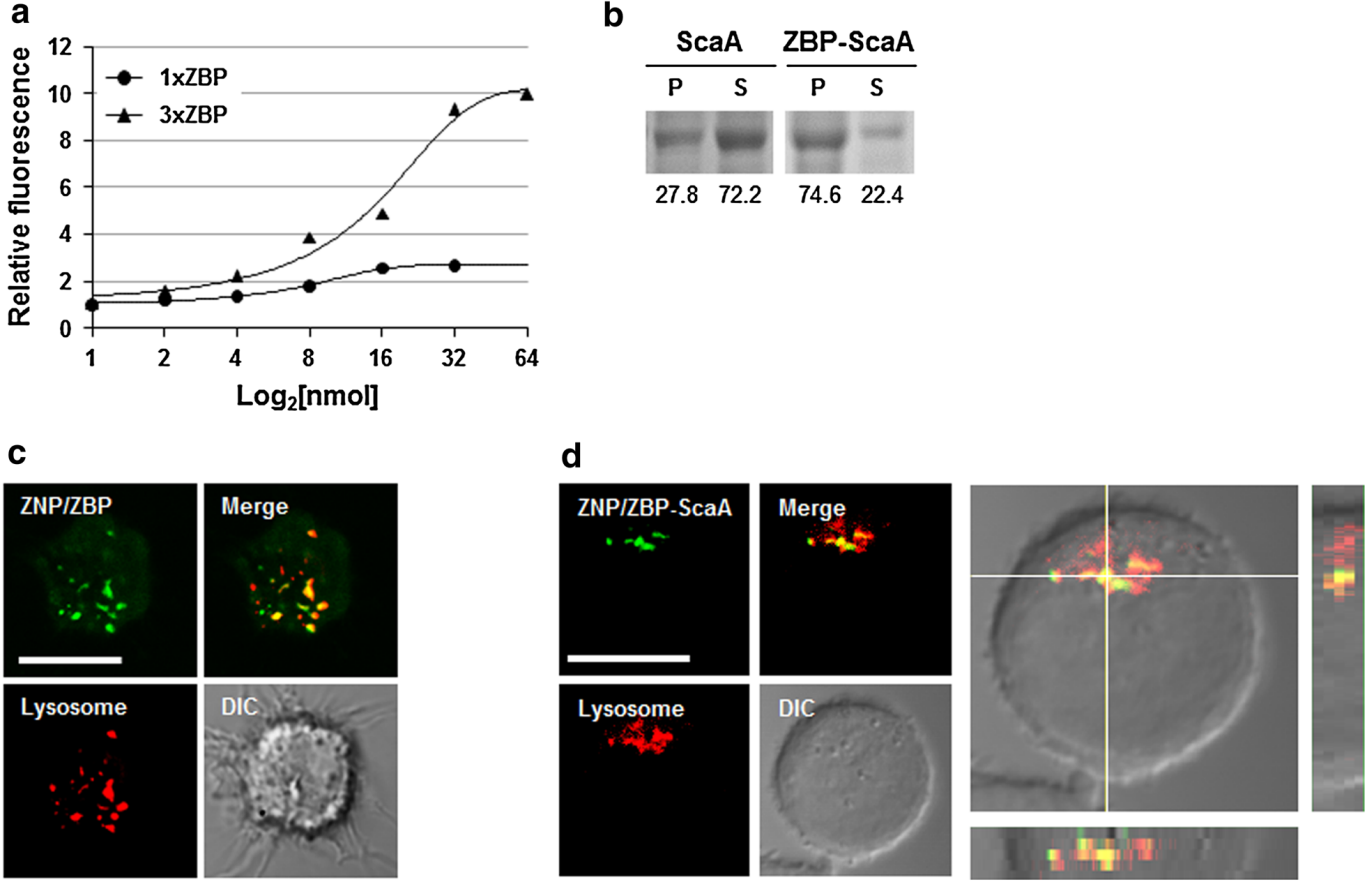
Complex formation of ZNP/ZBP and its delivery into DCs. **a** Relative affinity of 1× ZBP and 3× ZBP is assessed by measuring fluorescent intensity of ZNP-peptide complex. 50 μg of ZNPs were incubated with indicated amount of peptide and the fluorescent intensities of the complex were measured after washing with PBS. **b** Gel electrophoresis data showing the relative fraction of ZNP-bound (P, pellet) or unbound (S, supernatant) ScaA after incubation with ZNPs and ScaA with or without 3× ZBP fusion. Relative intensity of the protein bands (P and S) was indicated below. **c** Intracellular delivery of ZNP/ZBP complex into DCs was assessed by fluorescence confocal microscopy after incubation of DC2.4 cells with ZNP and 3× ZBP-FITC complex. Lysosomes (*red*) were stained with LysoTracker. **d** Intracellular delivery of ZNP/ZBP-ScaA complex into DCs was assessed by fluorescence confocal microscopy after incubation of DC2.4 cells with ZNP and 3xZBP-ScaA complex. ScaA antigens (*green*) and Lysosomes (*red*) were stained with anti-ScaA antibody and LysoTracker, respectively. Intracellular co-localization of ZNP/ZBP-ScaA complexes with lysosomes were assessed by confocal imaging of z-stacks and orthogonal views (*yz* and *xz*) were shown in *left* and *bottom* panels. *DIC* differential interference contrast. *White bar* 10 μm

To make use of ZNP as an antigen carrier, we first screened ZBPs from a random 8-mer peptide library and examined their affinity to ZNP. After three rounds of screening, the amino acid sequences of selected ZBPs were determined by mass spectrometry and the detection frequencies of amino acids in each position (P1–P4) from amino terminals are presented in Fig. 2a. Based on the detection frequency data, we synthesized eight peptide candidates for further assays to determine their affinity to ZNP. The synthesized peptides consisted of selected amino acids and a linker (GGDA) to allow for flexibility (Table 1) [2]. The relative affinity of the selected peptides to ZNPs was compared by measuring fluorescent intensity at 488 nm after binding assays using the FITC-labelled peptides. As shown in Fig. 2b, two peptides with sequences of FPYPGGDA and FPYDGGDA exhibited the highest affinity to ZnO nanoparticles among the eight peptides examined. The binding affinity of the ZBP, FPYDGGDA, to ZNP as determined by isothermal titration calorimetry (Fig. 2c), showed a *K*_*a*_ value of 2.26 × 10^6^ M^−1^. This is stronger than a previously reported peptide, RPHRKGGDA (*K*_*a*_ = 6.9 × 10^5^ M^−1^) [2], and comparable to that of EAHVMHKVAPRP (*K*_*a*_ ≤ 5.9 × 10^6^ M^−1^) [14]. The binding constant of this novel ZBP is comparable to those of strong inorganic binders, which have *K*_*a*_ values ranging from 1 × 10^4^ to 1 × 10^8^ M^−1^ [2, 31]. To further enhance the binding affinity of the peptide to ZnO, we generated a triplicate tandem repeat of the peptide (3× ZBP) [2]. This 3× ZBP showed enhanced binding to ZNP compared to the 1× ZBP (Fig. 3a). The binding of each peptide was saturated at ~16 nmol for 1× ZBP and ~32 nmol for 3× ZBP with 50 μg of ZNPs, resulting in 0.32–0.64 nmol of peptide binding per 1 μg of nanoparticle. Next, we generated ScaA antigen [28] fused with 3× ZBP for scrub typhus vaccine study. As expected, addition of 3× ZBP to the recombinant protein enhanced the binding of ScaA to ZNPs by ~2.5 fold over that of ScaA without 3× ZBP (Fig. 3b).

**Table 1:** Amino acid sequences of synthesized ZBPs

ID	Peptide sequences	M.W.
1	FPYPGGDA	1340.44
2	FPYDGGDA	1358.41
3	FQYPGGDA	1371.45
4	FQYDGGDA	1389.42
5	WPYPGGDA	1379.47
6	WPYDGGDA	1397.44
7	WQYPGGDA	1410.49
8	WQYDGGDA	1428.46

In order to investigate potential changes in colloidal properties of ZNPs upon the protein binding in aqueous solutions [32], the hydrodynamic diameters and zeta potential values for ZNPs were measured. As seen in Table 2, the hydrodynamic diameters of ZNPs were increased in aqueous solutions, especially in phosphate-buffered solution containing NaCl (150 mM), when compared to that in ethanol. The ionic solution shields the surface charge and may cause agglomeration [32]. Nevertheless, it is interesting to note that surface binding of ZBP-ScaA (isoelectric point = 6.57) on ZNPs stabilize the hydrodynamic diameters of ZNPs and reduced the negative charges. Surface coating of ZNPs with the protein antigen may reduce agglomeration and negative zeta potential, which may also enhance the cellular uptake of the complexes.

**Table 2:** Characterization of hydrodynamic diameters and zeta potentials of ZNPs

Samples (solvent)	Hydrodynamic diameter (nm)	Zeta (mV)
ZNP (EtOH)	75.18	13.6
ZNP (H_2_O)	200.3	−29.8
ZNP/ZBP (H_2_O)	232.6	−29.4
ZNP/ZBP-ScaA (H_2_O)	280.2	−19.8
ZNP (PBS)	460.1	−24.4
ZNP/ZBP (PBS)	552.1	−22.7
ZNP/ZBP-ScaA (PBS)	295.2	−12.7

Finally, we examined the intracellular delivery of ZNP coated with 3× ZBP labelled with FITC or 3× ZBP-ScaA conjugate. Consistent with our previous study [2], the peptides and protein antigen immobilized on ZNPs were efficiently delivered into the cytoplasm of DC2.4 cells and formed aggregates that primarily co-localized with lysosomes (Fig. 3c, d), indicating that ZNP complexes are internalized through phagocytosis [2].

### Protective immune responses against *O. tsutsugamushi* infection

**Fig. 4:**
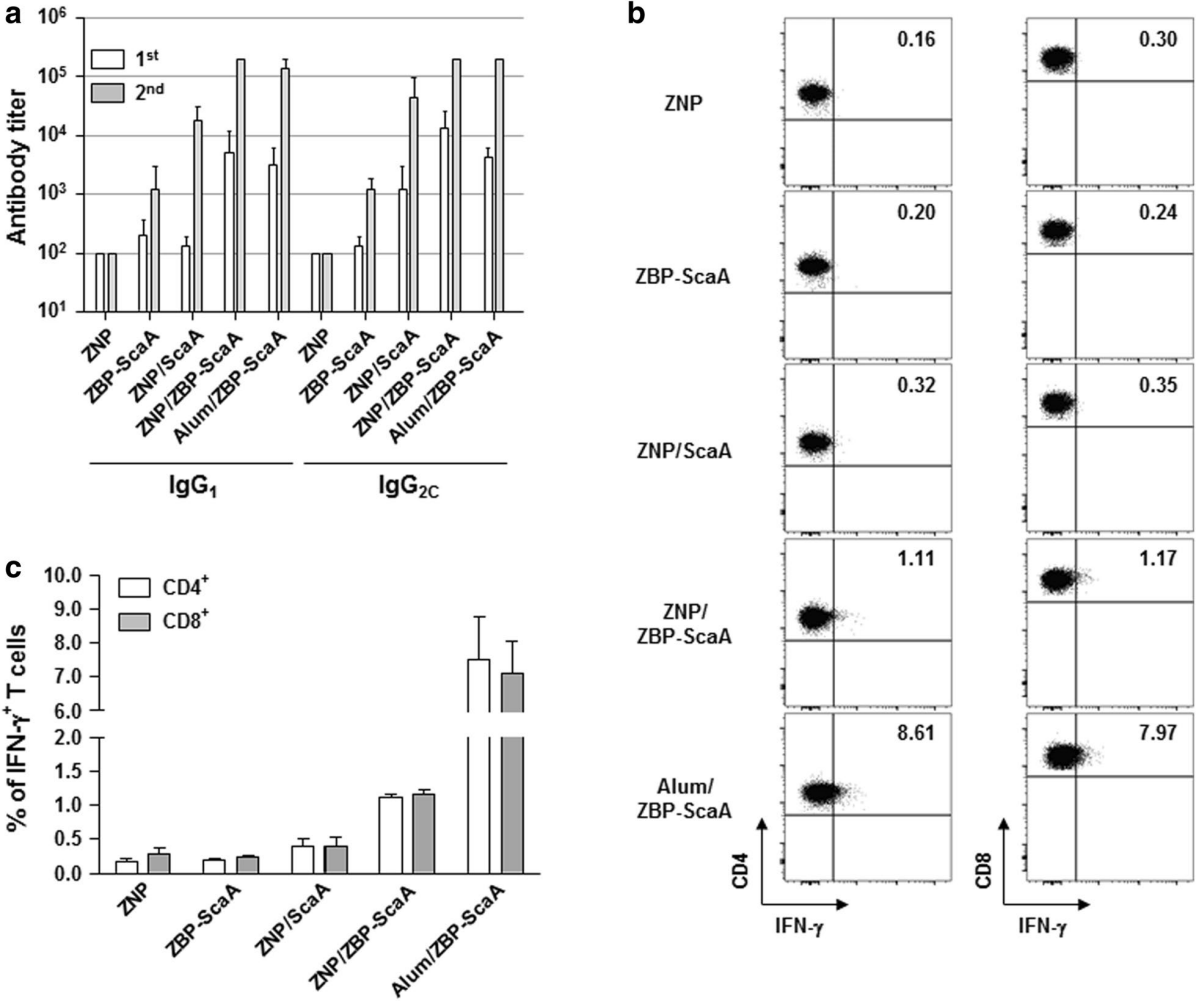
Induction of ScaA specific immune responses. **a** Antibody responses observed in the immunized mice. Mice were immunized with ZNP, ZBP-ScaA, ZNP/ScaA, NP/ZBP-ScaA, or Alum/ZBP-ScaA (*n* = 3/group) twice and the levels of anti-ScaA IgG_1_ and IgG_2C_ in the sera were measured by ELISA at 1 week after immunization. Antibody titer was assessed up to 102,400. **b** IFN-γ-positive CD4^+^ or CD8^+^ T cells in splenocytes stimulated with ScaA antigen were detected at 1 week after second immunization as in **a**. Representative *dot blots* obtained by FACS analysis were presented. **c** Average percentile of three independent experiments for IFN-γ-positive CD4^+^ or CD8^+^ T cells in splenocytes from immunized mice were presented. *Error bars* mean ± SD

**Fig. 5:**
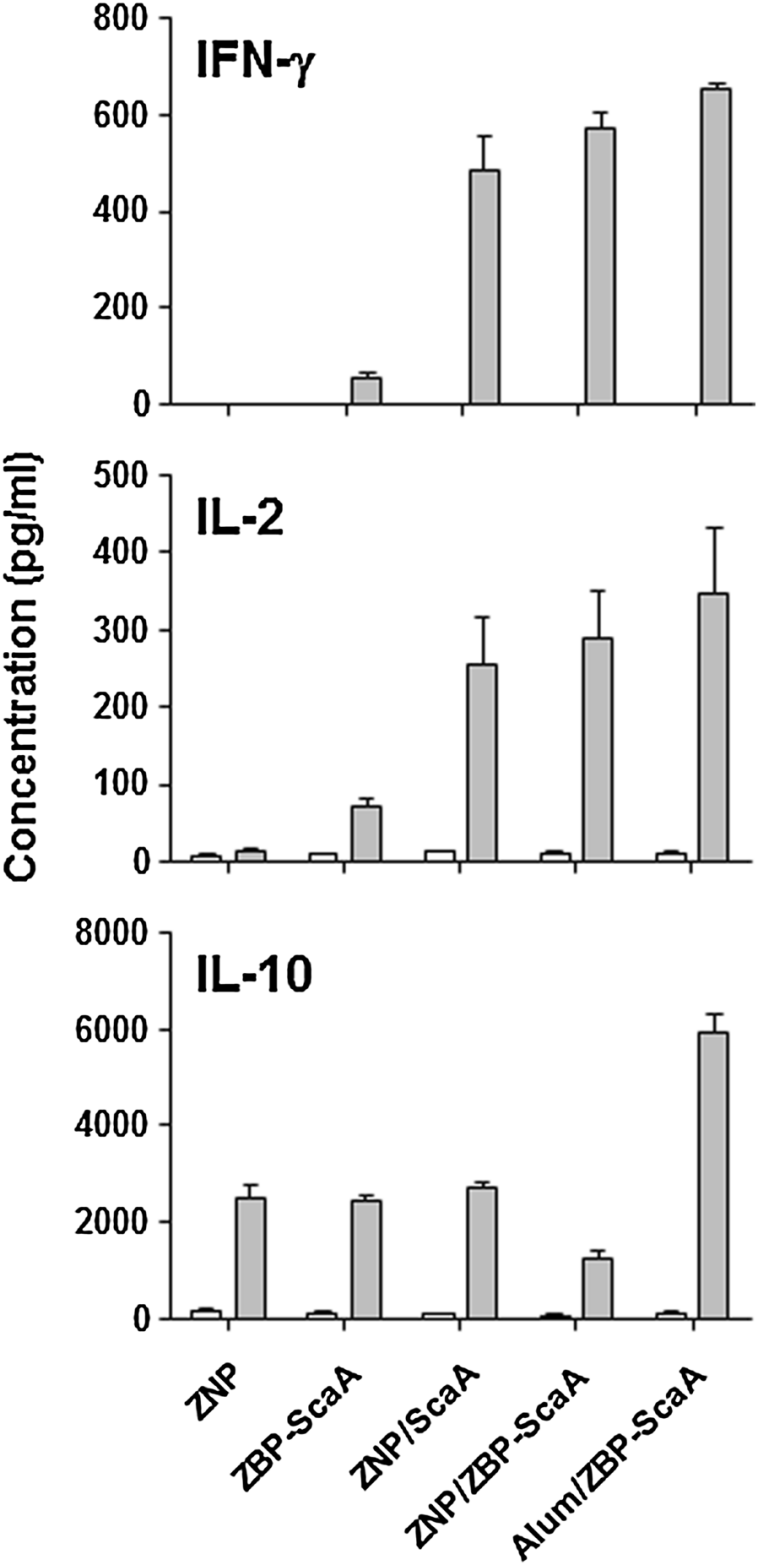
Production of type 1 (IFN-γ and IL-2) and type 2 (IL-10) cytokines from splenocyte cultures in the absence (*white bar*) or presence (*gray bar*) of ScaA antigen. Splenocytes were collected from mice (*n* = 3/group) immunized twice with the indicated antigens at 2 weeks after second immunization

Since systemic exposure to ZNPs complexed with ovalbumin (OVA) antigen could enhance antigen-specific immune responses including OVA-specific antibodies and T cells [18], we measured humoral immune responses in mice immunized with ScaA antigen immobilized on ZNPs. At 1 week after primary and secondary immunization, ScaA-specific antibodies were examined. As seen in Fig. 4a, remarkable increases of anti-ScaA IgG_1_ and IgG_2C_ antibodies in the sera were observed in mice immunized with ZNP/ZBP-ScaA compared with ZNP or ZBP-ScaA-treated mice. It is also notable that the antibody responses of the ZNP/ZBP-ScaA mice group were significantly higher than those of the ZNP/ScaA group, suggesting that enhanced binding of ZBP-ScaA to ZNPs via ZBP could further increase antigen-specific humoral immunity. In addition, the levels of ScaA-specific antibodies in the ZNP/ZBP-ScaA mice group were comparable to those of mice immunized with a conventional adjuvant, Alum (Alum/ZBP-ScaA). To investigate whether immunization of ZNP/ZBP-ScaA complexes can also induce cell-mediated immunity, we examined T cells responses by measuring their production of IFN-γ in an antigen-dependent manner (Fig. 4b, c). The frequencies of IFN-γ-secreting CD4^+^ or CD8^+^ T cells in spleens of NP/ZBP-ScaA-immunized mice significantly increased by approximately six or four fold, respectively, when compared with the non-immunized group (ZNP). In contrast, CD4^+^ or CD8^+^ T cells from spleens of other control groups (ZBP-ScaA or ZNP/ScaA) did not show significant IFN-γ secretion upon antigenic stimulation. Only marginal increases (about 2 fold) in IFN-γ^+^ production by CD4^+^ T cells from the ZNP/ScaA group were detected. Even though the relative frequencies of IFN-γ-secreting CD4^+^ or CD8^+^ T cell responses in ZNP/ZBP-ScaA-immunized mice is lower than those of the Alum/ZBP-ScaA group, these results clearly show that immunization of ZNP/ZBP-ScaA can induce antigen-specific T cell immunity as well as humoral responses in vivo. To expand these findings, we examined levels of signature cytokines for type 1 (IFN-γ and IL-2) and type 2 T cell responses (IL-10) in the culture media of splenocytes from immunized mice after stimulation with ScaA antigen (Fig. 5). Substantial production of type 1 cytokines (IFN- and IL-2) from the splenocytes in an antigen dependent manner was consistently observed in the immunized groups where ZNP or Alum was used as adjuvant. In addition, secretion of type 2 cytokine, IL-10, was relatively lower in the splenocytes from mice immunized with ZNP/ZBP-ScaA than other immunization groups, suggesting an immune bias toward T_H1_ responses by ZNP/ZBP-ScaA immunization.

**Fig. 6:**
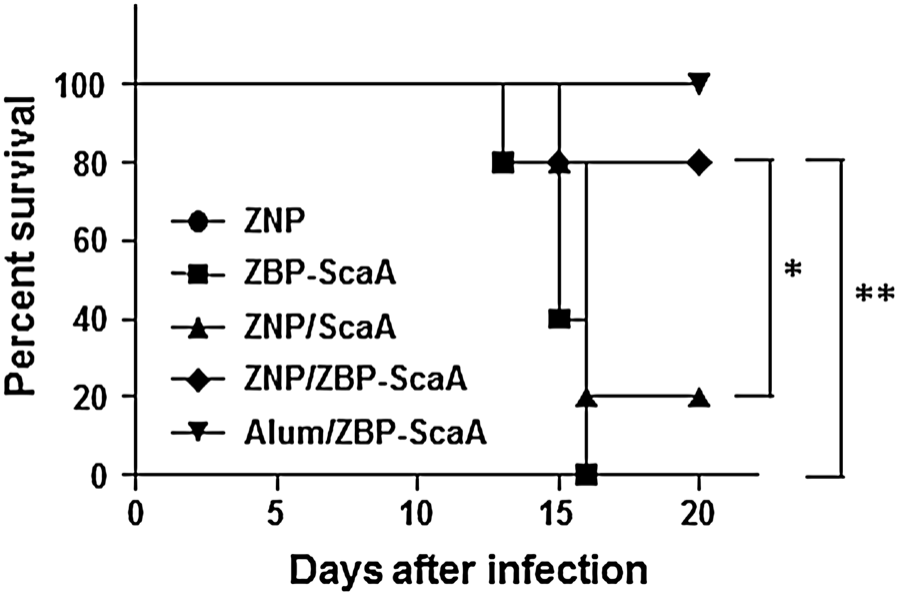
Increased survival of ZNP/ZBP-ScaA immunized mice. Mice (*n* = 5/group) were immunized with ZNP, ZBP-ScaA, ZNP/ScaA, ZNP/ZBP-ScaA, and Alum/ZBP-ScaA three times at 2 weeks interval. One week after last immunization, mice were challenged with 100 × LD_50_ of *O. tsutsugamushi*. Their survival was monitored until all the surviving mice recovered from the disease. *p < 0.05; **p < 0.01

Finally, the protective effect of ZNP/ZBP-ScaA immunization against *O. tsutsugamushi* infection was investigated in vivo by challenging mice with 100 × LD_50_ of *O. tsutsugamushi* at 1 week after the third immunization. As shown in Fig. 6, a significant level of protection against bacterial challenge was observed in the ZNP/ZBP-ScaA-immunized group as well as in Alum/ZBP-ScaA-immunized mice. In contrast, there was no significant protection in mice immunized with either ZNP/ScaA or ZBP-ScaA alone. Therefore, administration of ZBP-ScaA complexed with NP could provide protective immunity against *O. tsutsugamushi* infection as efficiently as an adjuvant-based immunization (Alum/ZBP-ScaA).

## Discussion

Soluble peptide and protein antigens alone are generally weak immunogens due to their inefficient delivery into antigen-presenting cells (APCs) and low immune-stimulatory nature. Particulation of a soluble antigen could facilitate antigen delivery into APCs, such as dendritic cells and macrophages, and enhance its immunogenicity. Costimulatory signals also need to be concomitantly induced when antigens are delivered into APCs in order to generate effective adaptive immune responses. In this study, we examined ZNP as a vaccine adjuvant by utilizing a ZBP for the particulation of a soluble antigen. We also investigated whether enhanced affinity of an antigen to ZNPs can induce stronger adaptive immunity when delivered as a complex.

First, we exploited physical properties of NPs which can selectively absorb proteins on their surfaces [33], thereby forming particulated forms of soluble antigens. Previously, metal oxide nanoparticles including ZNP were shown to bind multiple plasma proteins such as albumin, immunolglobulin, and fibrinogen [34]. The binding constant (*K*_*a*_) of albumin to naïve or polyethyleneimine-modified ZNP ranges from 2.6 × 10^4^ to 7.9 × 10^4^ M^−1^ [35]. A novel ZBP screened in this study showed almost a hundred times enhanced affinity (*K*_*a*_ = 2.3 × 10^6^ M^−1^) to ZNP when compared to that of serum albumin, and its affinity can be further increased by using tandem repeats of the ZBP sequence (Figs. 2, 3). Previously, we showed that conjugation of a tumor antigen with a high affinity ZBP could significantly enhance intracellular delivery of the antigen into dendritic cells after complex formation with Fe_3_O_4_–ZnO core–shell nanoparticles [2]. These complexes were primarily delivered into lysosomal compartments where the antigens can be processed for presentation by APCs to T cells. Intracellular delivery of ZBP-ScaA antigens complexed with ZNPs into the lysosomal compartments of a dendritic cell line was also confirmed in this study (Fig. 2c). Since the novel ZBP sequence (FPYDGGDA) selected in this study showed even stronger binding to ZNPs when compared to a previous ZBP sequence (RPHRKGGDA), we could expect more efficient delivery of the ZBP conjugated antigens into APCs when complexed with ZNPs. In addition, we found that coating of ZNPs with ZBP-ScaA antigens can reduce negative surface charge and particle agglomeration, especially in buffered salt solution, which may also facilitate intracellular uptake of the ZNP-antigen complexes [32, 36].

Second, ZNP itself can exert immunomodulatory and/or inflammatory effects on APCs, thereby promoting specific immune responses [37, 38]. ZNP has been shown to induce reactive oxygen species (ROSs), inflammatory cytokines and chemokines (IL-1β, IL-6, IL-12, TNF-α, CXCL-5, CXCL-9, and CXCL-10), and surface activation markers (MHCII, CD1d, CD11c, CD40, CD80, and CD86) in APCs. Induction of these innate immune responses may result from the direct recognition of ZNPs by Toll-like receptors (TLR4 or TLR6) [39, 40] or indirectly by intracellular ROS generation resulting from disrupted cellular zinc homeostasis [37, 38]. Recently, a study reported that particulate ZNPs might contribute to systemic inflammation in a TLR4-dependent manner when exposed to respiratory tracts [39]. Interestingly, among several inorganic NPs (TiO_2_, TiO_2_-silica, single-walled carbon nanotubes, multi-walled carbon nanotubes, and ZNPs), ZNP had the most drastic immunological effects, leading to high expression of IL-1β and CXCL-9 in APCs [41]. Considering that classical alum adjuvants have been shown to induce strong inflammatory responses by activation of inflammasome resulting in secretion of IL-1β, and by the release of danger-associated molecular patterns from the injured cells [42], the inflammatory and cytotoxic nature of ZNPs could be the basis of its capacity as an immuno-adjuvant. Previously, it was showed that peritoneal administration of OVA antigen with ZNP could significantly enhance OVA-specific IgG_1_ and IgE responses in the serum when compared to those of OVA-only sensitized mice after boost immunization [18]. The enhanced antibody responses after boost immunization as well as proliferation of lymphocytes upon antigenic stimulation indicate that ZNPs have an adjuvant effect for the induction of adaptive immunity. They also claim that the adjuvant effect of ZNP drives OVA-specific immune responses toward a T_H2_ response, as measured by increased IgG_1_ and IgE responses and by secretion of IL-4 and IL-5 in immune splenocytes upon OVA stimulation [18]. In this study, we found that both IgG_1_ and IgG_2C_ responses, representing T_H2_ and T_H1_ responses respectively, against ScaA antigen are dramatically enhanced by subcutaneous immunization of ZNP complexed with ZBP-ScaA antigen when compared to those of the ScaA or ZNP/ScaA immunized groups (Fig. 4a), suggesting the stronger affinity of the associated antigen to ZNP may further enhance the adjuvant efficacy of ZNP towards both T_H2_ and T_H1_ responses. Increased IFN-γ-positive T cell responses as well as reduced IL-10 production upon antigenic stimulation also further support the idea that the ZNP/ZBP-ScaA complex is more potent in generating T_H1_ response than ZNP/ScaA (Figs. 4, 5). Finally, we observed that immunization with the ZNP/ZBP-ScaA complex can provide significant protection of immunized mice from lethal challenges of *O. tsutsugamushi,* whereas ZNP/ScaA failed to do so (Fig. 5). Considering that both humoral and cell-mediated immune responses are required for efficient protection against bacterial challenge [28], we can conclude that the level of antibody and T cell responses against ScaA induced by immunization with ZNP/ZBP-ScaA were strong enough to protect the mice. Therefore, enhanced affinity of an antigen to ZNP can contribute to generating stronger adaptive immune responses when delivered together with ZNP.

Although the US Food and Drug Administration approved the use of ZNPs as food additives and sunscreen ingredients, safety concerns regarding its in vivo toxicity have persisted [38, 43]. Extensive studies on the in vivo toxicity of ZNPs have been performed in animal models after exposing ZNPs via skin, oral, and inhalation routes, which may cause ZNP accumulation in vital organs such as liver, kidney, and lungs as well as systemic increase of Zn^2+^ levels [38, 44]. Administration of excess ZNPs, ranging up to hundreds of mg/kg, via those routes can induce transient toxicity and inflammation in the affected organs, potentially due to dissolution of ZNPs and generation of ROS, but the excess ZNPs are not retained and are eventually eliminated from the system [38, 44]. In a few studies on the systemic toxicity of ZNPs or ZnO-containing NPs after parenteral administration [16, 45], transient accumulation of NPs, as well as local inflammation, in several internal organs or injection site have been observed. However, administered NPs were gradually removed from the body and the animals did not develop any significant systemic inflammation nor any detectable morbidity [16, 45]. In this study, we also did not observe any significant change with respect to morbidity, body weight, and food intake after subcutaneous injection of ZNPs. Therefore, ZNPs might be safe enough to be used as a vaccine adjuvant after parenteral administration, although prolonged monitoring is necessary to exclude the potential for long-term toxicity with repeated injection for boost immunization.

## Conclusions

In summary, ZNPs possess good adjuvant potential as a vaccine carrier when combined with an antigen having a high affinity to ZNPs. The immuostimulatory effect of ZNP as well as particulation of soluble antigen through complex formation might contribute to efficient delivery into antigen-presenting cells and concurrently enhance antigen-specific adaptive immune responses. In addition, the large surface area of NPs may allow for a dose-sparing effect for the vaccine antigen. Indeed, ZNPs complexed with ZBP-ScaA antigen could induce strong antibody responses as well as protective immunity against lethal challenges of *O. tsutsugamushi*. Therefore, application of ZNPs combined with a specific soluble antigen could be a promising strategy as a novel vaccine carrier system.

## Methods

### Synthesis of zinc oxide nanoparticles

ZNPs were synthesized using an ethanol solution containing 50 mM of Zn(NO_3_) 6H_2_O and 12 mM of cetyltrimethylammonium bromide (CTAB). After stirring for 1 h, an equivalent volume of a 0.1 M solution of NaOH in ethanol was injected. After dynamic stirring for 1 h again, the mixed solution was rinsed several times with ethanol using an ultrasonicator and a centrifuge. The resulting ZNPs were dispersed in ethanol.

### Screening of zinc oxide-binding peptides

Random eight-mer peptides were synthesized at a peptide synthesis facility (Peptron). 100 μg of peptide was incubated with 1 mg of ZNPs for 1 h at room temperature. The peptide-ZNP complexes were then washed 5 times with phosphate-buffered saline (PBS) and the bound peptides were resolved by 25% sodium dodecyl sulphate–polyacrylamide gel electrophoresis (SDS-PAGE). Peptide sequences on the gel were determined by Edman sequencing by a Procise 494 automated protein sequencer (Applied Biosystems).

### ZBP binding assay

To measure the relative affinity of ZBPs to ZNPs, peptides labeled at their carboxyl terminals with fluorescein isothiocyanate (FITC) was used. Different concentrations of FITC-tagged 1× or 3× ZBPs were incubated with 50 µg of ZNPs for 1 h at room temperature. ZBP-ZNP complexes were washed 5 times in PBS, resuspended in 100 µl of PBS, and then transferred to a 96 well black plate for fluorescence intensity measurement using Infinite^®^M200 PRO (Taken Switzerland). For ZBP-ScaA binding assay, purified 3× ZBP-ScaA or ScaA proteins were incubated with ZNPs for 1 h at room temperature. The bound proteins on ZNPs were collected by centrifugation and quantitated by Coomassie blue staining after SDS-PAGE.

### Isothermal titration calorimetry

The affinity between a selected ZBP and ZNPs was determined by isothermal titration calorimetry (ITC). ITC was performed using a MicroCal VP-ITC Microcalorimeter with Origin software and VPViewer2000 (MicroCal Inc.). Titrations were performed by injecting 30 consecutive 10 μl aliquots of ZBP peptide (0.2 mM) into the ITC cell (volume = 1.4301 ml) containing nanoparticles (1 μM). Titration experiments were performed at 25 °C to determine the binding constant of ZBPs to the nanoparticle. The ITC data were corrected for the heat of dilution of the titrant by subtracting mixing enthalpies for 10 μl injections of ZBPs into nanoparticle-free solution. Binding stoichiometry, enthalpy, entropy, and equilibrium association constants were determined by fitting the corrected data to a bimolecular (One type of binding site) interaction model (Microcal Origin software version 7.0).

### Measurement of colloidal properties of ZNP

Microstructure of the ZNP was analyzed by transmission electron microscopy (TEM, FEI Tecnai F20) operated at an accelerating voltage of 200 kV where samples were prepared on carbon coated copper mesh grids. From the TEM image, about 80 ZNPs were counted to get the size distribution followed by Gaussian fitting to estimate an average diameter. Optical property of the ZNPs dispersed in ethanol was determined by employing a photoluminescence spectrometer (PL, Shimadzu RF-5300 PC). Hydrodynamic diameters and surface charges of ZNPs, before and after surface modification, were monitored by dynamic light scattering (DLS) and zeta potential measurements (Malvern Nano-ZS90). Samples for DLS and zeta potential measurements were prepared in deionized (DI) water or phosphate-buffered saline (PBS). Prior to measurements, 1–2 ml of the samples were filled in disposable cells after gentle sonication.

### Immunofluorescence assay

Immunofluorescence confocal microscopy was used to visualize *O. tsutsugamushi* [46] and ZNP/ZBP complexes taken up DC2.4 cells. L929 cells were cultured on 12-mm diameter glass coverslips in 24-well plates and inoculated with *O. tsutsugamushi*. Plates were spun at 500×*g* for 5 min to synchronize bacterial contact with the host cell monolayers and then incubated at 37 °C for the indicated times. Intracellular bacteria were stained by differential immunofluorescence as previously described [28]. DC2.4 cells taken up ZNP/ZBP or ZNP/ZBP-ScaA complexes were stained with LysoTracker Red DND-99 (Molecular probe) to examine the co-localization of the complexes with lysosomes. Cells were observed using an Olympus FV1000 laser confocal microscope (Olympus) and analyzed using the Fluoview software (Olympus).

### Production of recombinant ScaA antigens

The ScaA_88–3000_ protein (GenBank accession no. AM494475.1) was purified from *E. coli* as described previously [28]. To produce recombinant ZBP-ScaA protein, annealed double-stranded DNA (5′- GATCCTTTCCGTATGATGGCGGCGATGCGTTTCCG TATGATGGCG GCGATGCGTTTCCGTATGATGGCGGCGATGCGG-3′, BamHI and EcoRI sites underlined) encoding 3× ZBP (FPYDGGDAFPYDGGDAFPYDGGDA) was cloned into the pET28a-ScaA plasmid [28] after digestion with BamHI and EcoRI. The recombinant protein was also produced and purified from *E. coli* as described previously [28]. The purified proteins were treated with endotoxin removal columns (Pierce) before use. Endotoxin contamination of the purified recombinant proteins was determined using QCI-1000^®^ End-Point Chromogenic Endotoxin Detection kit (Lonza) and less than 20 E.U./ml.

### Preparation of *Orientia tsutsugamushi*

*Orientia tsutsugamushi* Boryong strain was purified using a modified Percoll gradient purification method [47]. *Orientia tsutsugamushi* was propagated in L929 cells. At 3–4 days post-infection, infectivity was determined using an indirect immunofluorescence assay [28]. When an infection rate of >90% was achieved, the cells were harvested by centrifugation at 6000×*g* for 20 min. The cell pellet was resuspended with Tris-sucrose (TS) buffer (33 mM Tris–Cl (pH 7.4) and 0.25 M sucrose) and homogenized using 100 strokes of a Polytron homogenizer (Wheaton Inc.) followed by centrifugation at 200×*g* for 5 min. The supernatant was then mixed with 40% Percoll (Pharmacia Fine Chemicals) in TS buffer and centrifuged at 25,000×*g* for 60 min. The bacterial band was collected and centrifuged at 77,000×*g* for 30 min. The bacterial pellet was washed 3 times in TS buffer, resuspended in Dulbecco’s Modified Eagle Medium (DMEM), and stored in liquid nitrogen until use. The infectivity titer of the inoculum was determined as previously described [47].

### Flow cytometry analysis

Lymphocytes collected from the spleens of immunized mice were cultured for 18–20 h in RPMI 1640 medium (Gibco) supplemented with 10% heat-inactivated FBS, 50 nM β-mercaptoenthanol, 100 units/ml penicillin/streptomycin, 2 mM l-glutamine (Welgene), and 10 μg/ml of purified ScaA in 24-well, flat-bottomed culture plates (5 × 10^6^ cells/well). After incubation, GolgiPlug (BD Biosciences) was added for 6 h. Lymphocytes were washed three times with ice-cold FACS buffer (PBS containing 1% bovine serum albumin and 1 mM EDTA). Cells were blocked on ice for 30 min with ultra-block solution containing 10% rat sera, 10% hamster sera, 10% mouse sera (Sigma) and 10 μg/ml of 2.4G2 monoclonal antibody (BD Pharmingen). Cells then stained with FITC-conjugated CD4 or PE.cy7-conjugated CD8 antibodies (BD Pharmingen) for 30 min at 4 °C. After surface CD4 or CD8 staining, cells were washed three times with ice-cold FACS buffer and subjected to intracellular cytokine staining using the Cytofix/Cytoperm kit according to the manufacturer's instructions (BD Biosciences). Intracellular interferon-γ (IFN-γ) was stained using an APC-conjugated anti-IFN-γ antibody (BD Pharmingen) for 30 min at 4 °C. The stained cells were analyzed with a FACS LSRII flow cytometer (BD Biosciences). Data were analyzed by FlowJo software version 8.8.6 (FlowJo).

### Enzyme-linked immunosorbent assay (ELISA)

To determine the titer of antibodies specific to ScaA in the sera of immunized mice, immunoassay plates (Nunc) were coated with 100 μl of purified antigen at a concentration of 1 μg/ml at 4 °C overnight. The plates were then blocked for 2 h at room temperature with PBS containing 5% skim milk. 100 μl of serum samples serially diluted twofold were incubated for 2 h at room temperature. After washing with PBS containing 0.05% Tween20 (PBST), horseradish peroxidase (HRP)-conjugated goat anti-mouse IgM, IgG_1_, or IgG_2c_ (Santa Cruz Biotechnology) was added and incubated for 2 h at room temperature. Wells were washed with PBST and incubated with 3,3′,5,5′-tetramethylbenzidine (TMB) peroxidase substrate solution (KPL) for 7 min. The reactions were stopped by addition of 1 M phosphoric acid solution. Absorbances were measured at 450 nm using a microplate reader (Beckman Coulter Inc.).

### Cytokine analysis

A total 10^6^ splenocytes collected from immunized mice was cultured in the absence or presence of 10 μg/ml of purified ScaA antigen for 24 h at 37 °C, and supernatant was analyzed for IFN-γ, IL-2, and IL-10 by ELISA (eBioscience).

### Immunization of mice and challenges

Six to eight-week-old female C57BL/6 mice (Orient Bio Inc) were immunized (*n* = 5/group) subcutaneously three times at 2 week intervals. 20 μg of ScaA or ZBP-ScaA protein were incubated with 100 μg of ZNPs for 1 h at room temperature before immunization. Control immunization was performed with ScaA alone or using 2% Alhydrogel adjuvant (Invitrogen) at an adjuvant: protein solution volume ratio of 1:1 according to the manufacturer’s instruction. Blood samples were collected with retro-orbital puncture at 1 week after each injection and used to determine the serum antibody titer. One week after the final immunization, mice were challenged intraperitoneally with 10 × LD_50_ of *O. tsutsugamushi* Boryong strain. Mice survival was monitored for 1 month after bacterial challenge.

### Statistical analysis

Data was analyzed using the Graph Pad Prism 5.01 software (GraphPad Software) and SigmaPlot (Jandel). Statistical analysis was performed using the two-tailed Student’s *t* test with 95% confidence interval or One-way ANOVA. Data are expressed as the mean ± standard deviation. Statistical analysis on survival rates were performed using the Mantel-Cox Log Rank test. *p* value less than 0.05 was considered statistically significant.
